# Psychiatric diagnoses in 3275 suicides: a meta-analysis

**DOI:** 10.1186/1471-244X-4-37

**Published:** 2004-11-04

**Authors:** Geneviève Arsenault-Lapierre, Caroline Kim, Gustavo Turecki

**Affiliations:** 1 McGill Group for Suicide Studies, Douglas Hospital Research Centre, Department of psychiatry, McGill University, Montreal, Canada

## Abstract

**Background:**

It is well known that most suicide cases meet criteria for a psychiatric disorder. However, rates of specific disorders vary considerably between studies and little information is known about gender and geographic differences. This study provides overall rates of total and specific psychiatric disorders in suicide completers and presents evidence supporting gender and geographic differences in their relative proportion.

**Methods:**

We carried out a review of studies in which psychological autopsy studies of suicide completers were performed. Studies were identified by means of MEDLINE database searches and by scanning the reference list of relevant publications. Twenty-three variables were defined, 16 of which evaluating psychiatric disorders. Mantel-Haenszel Weighted Odds Ratios were estimated for these 16 outcome variables.

**Results:**

Twenty-seven studies comprising 3275 suicides were included, of which, 87.3% (SD 10.0%) had been diagnosed with a mental disorder prior to their death. There were major gender differences. Diagnoses of substance-related problems (OR = 3.58; 95% CI: 2.78–4.61), personality disorders (OR = 2.01; 95% CI: 1.38–2.95) and childhood disorders (OR = 4.95; 95% CI: 2.69–9.31) were more common among male suicides, whereas affective disorders (OR = 0.66; 95% CI: 0.53–0.83), including depressive disorders (OR = 0.53; 95% CI: 0.42–0.68) were less common among males. Geographical differences are also likely to be present in the relative proportion of psychiatric diagnoses among suicides.

**Conclusions:**

Although psychopathology clearly mediates suicide risk, gender and geographical differences seem to exist in the relative proportion of the specific psychiatric disorders found among suicide completers.

## Background

Suicide is an important public health problem that is among the leading causes of death in Western countries [1]. Over the last years, the relationship between suicide and mental disorders has been the focus of several studies and has generated important debate [2]. This relationship has been investigated by different strategies, but particularly by the psychological autopsy method [3], which is generally considered the method of choice to retrieve postmortem information on psychopathology. The psychological autopsy procedure entails the retrospective psychiatric assessment of the deceased by variable methodologies, but generally by means of proxy-based interviews. This procedure is also frequently completed by having access to medical and other relevant dossiers from the subject on whom the psychological autopsy is carried out [4,5].

It is well established that psychopathology is an important predictor of suicide completion [6], but there is considerable variability between studies in rates of total and specific psychiatric disorders [7]. One of the most consistent findings in suicidology is the excess of male suicides observed in most countries [8], with a few notable and important exceptions, such as China [1,9]. Geographic origin is another important source of variation [1]. However, the possibility that clinical and other behavioural factors could at least partly mediate gender and geographic differences in suicide rates has been little explored. The aim of this study was to carry out quantitative syntheses of overall and specific psychiatric diagnoses found in suicide studies and to explore possible gender and geographical differences in the distribution of psychiatric disorders among suicide completers.

## Methods

### Study identification

To identify studies for this review, the National Library of Medicine (NLM) PubMed database was searched up to December 2002 using English language and human study limits. The Medical Subject Heading (MeSH) terms "suicide AND psychological autopsy", "suicide AND psychopathology", "suicide AND (postmortem diagnoses OR postmortem diagnosis)", and "(mental disorders/*epidemiology) AND prevalence AND ((suicide/*statistics & numerical data) NOT suicide attempts)" were used. Finally, in order to find other articles not obtained through electronic searches, reference lists from original studies as well as from not independent studies were screened.

### Study selection

The inclusion criteria for considering articles for this review were as follow. Studies had to: 1) be original, 2) be published in English, 3) contain information on diagnostic distribution, 4) include suicide completers unselected according to specific mental disorders, 5) use of a psychological autopsy method, which for the purpose of this review was considered as the process of reconstructing psychiatric diagnoses based either on interviews with informants (regardless of the specific diagnostic instrument methodology) or on review of multiple official records that contained interviews with informants such as general practitioners, other professionals and relatives or friends, 6) use of standard diagnostic criteria (any versions of the Diagnostic and Statistical Manual of Mental Disorders, the International Classification of Diseases or the Research Diagnostic Criteria).

Studies were excluded if: 1) their sample was not independent from that investigated in another study (see below for criteria on which one was included), 2) they were reports on suicide in one specific diagnostic category and 3) if diagnoses were simply extracted from medical records without review of multiple sources of information.

A single reviewer (G.A.L.) made a prior screening to identify and select articles. When titles and abstracts were deemed adequate or when they remained too obscure to reach a verdict, full texts were retrieved for further evaluation in conformity with the inclusion and exclusion criteria.

### Study assessment

A total of 23 variables were defined, three of which relate to demographic information, four other concern the method of diagnosis, and 16 evaluate the presence of psychiatric diagnoses. To obtain the latter 16 variables (shown in table 1; see additional file), every diagnostic term used in the original studies was categorised into one of the 16 pre-defined groups. So diagnoses such as "intermittent depressive disorder" or "neurotic depression" reported in some studies were coded under "depressive disorders' variable and diagnoses such as "alcohol use", "alcohol misuse" and "alcohol abuse" were coded as "alcohol problems". All substances noted as other than alcohol were coded under "other substances problems". These two variables were then recoded as "any substance problems". The same was achieved with the "depressive disorders" and "bipolar disorders" which were recoded as "any affective disorders".

Disorders labelled as "other" or as a subset of various disorders without further specification were left aside. For all studies the most specific diagnosis was considered. That is, when the authors broke down general diagnosis such as "affective disorder" into "depressive disorders" and "bipolar disorders", only these more specific diagnoses were noted and accounted for in our study.

When two studies or more were carried on the same population, the study with the largest sample and the most informative report was consistently selected. When multiple diagnoses and principal diagnoses (those deemed by the investigators as more related to the suicide) were reported, preference was given to the former. In four cases, secondary diagnoses were added to principal diagnoses to obtain multiple diagnoses [10-13]. Studies for which controls were selected among psychiatric in-patients or matched to suicides by mental diagnosis, only suicide cases were included in our analysis [12,14]. In the study by Graham and Burvill [15], controls were older suicide completers, and so they were included in our suicide group. In the study by Hawton et al. [10], only diagnoses for suicides obtained by means of an interview were included. In three case-control studies [16-18], not all suicide cases were matched to a control. In these cases, we considered the full suicide sample in the descriptive analyses, but only the control-matched suicides in the quantitative analyses.

### Statistical analysis

Descriptive analyses and homogeneity tests were carried out before pooling the data. In order to determine the risks of having had a disorder, suicides and controls were recorded in 2 × 2 tables. These data were then stratified by the 16 outcome variables and Mantel-Haenszel Weighted Odds Ratios (OR) and 95% confidence intervals (95% CI) were estimated. Gender differences were also explored by means of Odds Ratios. Major disorders were then compared between the different demographic areas by means of χ^2 ^to assess variations in the diagnostic distribution across these demographic areas. All statistical analyses were carried out using Epi Info 6, version 6.04d (C.D.C., U.S.A.; W.H.O., Geneva, Switzerland).

## Results

A total of 152 studies were initially identified. After selection according to inclusion/exclusion criteria, 27 studies were included in this review. The most common reasons for exclusion were that a) no diagnostic distribution was provided (n = 46) [6,19-63], b) samples were pre-selected according to a psychiatric disorder (n = 30) [64-93], c) there was another report on the same sample that either included more subjects or was more informative (n = 29) [3,94-121]. Four other studies were about non-completers [122-125]. Another was not in English [126], and others reported only on one type of disorder [127,128], and therefore, they were all excluded. Additional 14 studies [7,129-141] were excluded because the diagnostic criteria were either unspecified or not standard.

The studies by Rich et al. [99] and by Foster et al [142] were not independent from, respectively, Rich et al. [143] and Foster et al. [144]. Although non-independent, these studies provided information of different quality, and thus, were included in our review. Accordingly, Rich et al. [99] and Foster et al. [142] were considered, respectively in the gender difference analysis and the case-control comparisons, whereas the study by Rich et al. [143] and Foster et al. [144] were considered for the descriptive analysis.

### Methodological assessment

Among the 27 studies that were retained, 52% (14/27) were case-control studies. Eighty-one percent (22/27) of the studies were published after 1990. Sixty-seven percent of the studies (18/27) used DSM diagnostic criteria, whereas only 22% (6/27) and 11% (3/27) used the ICD and RDC diagnostic criteria respectively. Multiple diagnoses were investigated in 63% (17/27) of the studies, whereas principal diagnoses only were given for the other 10 studies. A description of the demographic and methodological features of these 27 studies is shown in table 2.

**Table 2:** Description of the 27 studies included in this meta-analysis

Study	Year	Origin	Diagnostic criteria	Methods	Number of diagnoses	n Suicide	With a Dx (%)	n Control	with a Dx (%)	Matched
Appleby et al.[151]*	1999	England	ICD-10	Official records and interviews	Multiple	84	76 (90%)	64	17 (27%)	Living ± 5 year and sex
Apter et al.[145]*	1993	Israel	DSM-III	Official records and interviews	Principal	43	35 (81%)			
Asgard U.[147]*	1990	Sweden	RDC	Official records and interviews	Principal	104	99 (95%)			
Cavanagh et al.[14]	1999	Scotland	DSM-III	Official records and interviews	Principal	45	44 (98%)			
Cheng et al.[16]*	1995	Taiwan	DSM-III-R	Official records and interviews	Multiple	116	114 (98%)	226	130 (58%)	Living ± 5 years, sex, area of residence
Conwell et al.[156]*	1996	USA	DSM-III-R	Official records and interviews	Multiple	141	127 (90%)			
Foster et al.[142,144]	1997/1999	Ireland	DSM-III-R	Official records and interviews	Multiple	118	106 (90%)	117	30 (26%)	List of deceased's GP Age, gender, marital status
Harwood et al.[17]*	2001	England	ICD-10	Official records and interviews	Multiple	100	93 (93%)	54	N/A	Natural deaths Age and sex
Hawton et al.[10]	2002	England	ICD-10	Official records and interviews	Multiple	42	38 (90%)	84	6 (7%)	Living nurses ± 10 years, specialty and seniority
Henriksson et al.[11]	1993	Finland	DSM-III-R	Official records and interviews	Multiple	229	225 (98%)			
Houston et al.[12]	2001	England	ICD-10	Official records and interviews	Multiple	47	40 (85%)			
Lesage et al.[150]	1994	Canada	DSM-III-R	Official records and interviews	Multiple	75	69 (92%)	75	N/A	Living Neighbourhood, age, marital status and occupation
Phillips et al.[9]*	2002	China	DSM-IV	Interviews with informants	Principal	519	325 (63%)	536	93 (17%)	Accidental deaths Geographical areas
Rich et al.[143]	1986	USA	DSM-III	Official records and interviews	Multiple	283	258 (91%)			
Runeson B.[153]	1989	Sweden	DSM-III-R	Official records and interviews	Principal	58	57 (98%)			
Shaffer et al.[18]*	1996	USA	DSM-III	Official records and interviews	Multiple	119	108 (91%)			
Shaffi et al.[13]*	1988	USA	DSM-III	Official records and interviews	Multiple	21	20 (95%)	21	11 (52%)	Living friends Sex, age, race, education, religion, income, and father's education
Vijayakumar et al.[159]*	1999	India	DSM-III-R	Official records and interviews	Principal	100	88 (88%)	100	14 (14%)	Living SES, sex and ± 2 years
Waern et al.[154]*	2002	Sweden	DSM-IV	Official records and interviews	Multiple	85	82 (96%)	153	28 (18%)	Living Sex, ± 2 years
Boardman et al.[152]	1999	England	ICD-10	Multiple official records	Multiple	212	151 (71%)	212	40 (19%)	Unnatural deaths ± 5 years and sex
Cantor et al.[157]	1989	Australia	DSM-III-R	Multiple official records	Principal	47	41 (87%)			
Groholt et al.[149]*	1997	Norway	DSM-III-R	Multiple official records	Multiple	121	90 (74%)			
Thacore et al.[158]	2000	Australia	ICD-9	Multiple official records	Principal	75	46 (65%)			
Graham et al.[15]	1992	Australia	DSM-III	Multiple official records	Multiple	136	120 (88%)			
Brent et al.[148]	1999	USA	DSM-III	Interviews with informants	Multiple	140	115 (82%)	131	32 (24%)	Living Age, race, gender, country and SES
Cerel et al.[155]	2000	USA	RDC	Interviews with informants	Multiple	15	13 (87%)	201	70 (35%)	Non-suicide bereaved family
Arato et al.[146]*	1987	Hungary	RDC	Interviews with informants	Principal	200	162 (81%)			

* Based on axis I disorders only.

N/A – information not available or not clear

### Demographic features

A total of 3275 suicides were included in our study with a mean number of 121 (standard deviation (SD) 103) suicides per study. There were 11 studies where diagnoses were given by gender for a subtotal of 933 males and 462 females [10,11,18,99,144-150]

There were 14 studies [10-12,14,17,142,145-147,149,151-154] carried out in Europe, including one in Israel [145]. These 14 European studies comprised a total of 1488 suicides. Seven studies were from North America [13,18,143,148,150,155,156] with 794 suicides, three others were from Australia [15,157,158] with 258 suicides and, finally, three were from Asia [9,16,159]. with 735 suicides.

### Diagnostic distribution

The mean percentage of suicides with a psychiatric diagnosis was 87.3 % (SD 10.0 %). However, only 14 of the 27 studies reported both axes I and II disorders (see table 2). The remaining 13 studies only assessed axis I diagnoses. The mean percentage of controls with a diagnosis was, as expected, lower (34.9 % SD 25.1 %). As a comparison, among studies not included because the diagnostic criteria were not specified or not standard, the mean percentage of suicides with a diagnosis was not statistically different from that of the studies included in this review (78.7% SD 21.0%, χ^2 ^: 2.27, p = 0.13).

On average, 43.2% (SD 18.5%) of suicide cases were diagnosed with any affective disorders (including depressive and bipolar disorders) and 25.7% (SD 14.8%) with other substance problems. In these groups, respectively, depressive disorders and alcohol problems were the most frequent. Finally, personality disorders represented 16.2% (SD 8.6%) of the suicide diagnoses and psychotic disorders, including schizophrenia accounted for 9.2% (SD 10.2%).

The samples from the 14 case-control studies were found homogeneous for the 16 outcome variables according to a homogeneity test (results not shown), allowing us to pool the individual studies and determine overall risks.

Table 1 (see additional file) shows that, with the exception of organic disorders and adjustment disorders, suicide cases had a higher risk of being diagnosed than controls with each of the diagnoses considered. Of these diagnoses, the risks for psychotic disorders were the highest (OR = 15.38; 95% CI: 3.53–97.82) followed by the variable "at least one psychiatric disorder" (OR = 10.50; 95% CI: 9.60–13.56). The risk for schizophrenia was also particularly high (OR = 5.56; 95% CI: 3.12–10.24). This is due to the fact that there were only 15 control subjects altogether diagnosed with schizophrenia and two with psychotic disorders.

Statistically significant differences were found when male and female suicide cases were compared (see table 3). However, gender-based comparisons should be considered cautiously as, when available, demographic information indicated that female suicides included in the studies reviewed tended to be older than males (table 5). Nevertheless, even considering this potential limitation, the results are interesting. The risks for alcohol (OR = 2.19; 95% CI: 1.63–2.95), other substance problems (OR = 2.02; 95% CI: 1.32–3.10), and any substance problems (OR = 3.58; 95% CI: 2.78–4.61), personality disorders (OR = 2.01; 95% CI: 1.38–2.95) or childhood disorders (OR = 4.95; 95% CI: 2.69–9.31) were greater in male as opposed to female suicides. On the other hand, the risks of having depressive disorders (OR = 0.53; 95% CI: 0.42–0.68) or any affective disorders (OR = 0.66; 95% CI: 0.53–0.83) were lower in males.

**Table 3:** Odds Ratios for major outcome variables across sexes

Disorders	n for females	n for males	OR (95% CI)	χ^2^	p-value
Any psychiatric disorders	398	801	0.98 (0.70–1.36)	0.02	0.881
Schizophrenia	17	44	1.30 (0.71–2.39)	0.79	0.373
Other psychotic disorders or psychosis NOS	15	40	1.33 (0.71–2.56)	0.88	0.347
Somatoform, anxiety and neurotic disorders	33	83	1.27 (0.85–1.97)	1.24	0.265
Bipolar disorders	26	43	0.81 (0.48–1.38)	0.68	0.409
Organic disorders	6	15	1.24 (0.45–3.60)	0.20	0.656
Adjustment disorders	31	64	1.02 (0.64–1.64)	0.01	0.917
*Disorders more likely if male*					
Alcohol problems	73	272	2.19 (1.63–2.95)	29.57	0.000
Other substances problems	32	122	2.02 (1.32–3.10)	11.89	0.001
Any substances problems	110	436	3.58 (2.78–4.61)	110.18	0.000
Personality disorders	41	153	2.01 (1.38–2.95)	14.60	0.000
Childhood disorders	13	117	4.95 (2.69–9.31)	34.57	0.000
*Disorders more likely if female*					
Depressive disorders	199	268	0.53 (0.42–0.68)	28.56	0.000
Any affective disorders	272	454	0.66 (0.53–0.83)	12.91	0.000
Other disorders	16	12	0.36 (0.16–0.82)	7.44	0.006

**Table 5:** Descriptive analysis of the age and sex of subjects

	**Age (mean ± SD)**	**n [Studies]**
All regions		
♂	28.5 ± 12.8	880 [11,18,143,145,148-150,158]
♀	34.5 ± 17.8	333 [11,18,143,147-149,158]
Both sexes*	41.6 ± 17.8	794 [11,14,17,149,151,154,157,158]
American Studies		
♂	26.0 ± 12.3	491 [18,143,148,150]
♀	27.3 ± 18.9	127 [18,143,148]
Both sexes*	N/A	N/A
European Studies		
♂	27.2 ± 15.4	314 [11,145,149]
♀	37.9 ± 18.9	191 [11,147,149]
Both sexes*	42.3 ± 20.8	672 [11,14,15,17,151,154]
Australian Studies		
♂	42.5	491 [158]
♀	45.7	15 [158]
Both sexes*	39.5 ± 5.2	122 [157,158]
Asian Studies		
♂	N/A	N/A
♀	N/A	N/A
Both sexes*	N/A	N/A

N/A – information not available

* Both sexes refers to studies in which information on age by sex was not provided, and thus, only mean age for the whole sample was available.

Analysing the data according to geographic areas, the diagnostic distribution of the key diagnoses found in suicides differed significantly between world regions (see table 4), but as mentioned above, potential age-related biases may apply (table 5). The American suicides were more often diagnosed with a psychiatric disorder than suicides in the other regions of the world; 89.7 % (SD 4.2 %) of the American suicides had at least one diagnosis, whereas 88.8 % (SD 8.9 %) of the European suicides, 83.0 % (SD 18.4 %) of the Asian suicides and 78.9 % (SD 15.3 %) of the Australian suicides had at least one psychiatric diagnosis.

**Table 4:** Diagnostic distribution across different regions of the world

	European (%)	North American (%)	Australian (%)	Asian (%)	χ^2^
Affective disorders	753 (48.5)	390 (33.6)	71 (32.7)	335 (51.3)	11.3*
Substances-related disorders	390 (18.6)	573 (40.1)	106 (24.1)	135 (26.7)	12.1*
Schizophrenia and other psychotic disorders or psychosis NOS	125 (7.5)	42 (4.2)	29 (24.3)	53 (8.4)	24.1*
Personality disorders	197 (16.8)	75 (13.4)	75 (17.7)	20 (17.7)	1.2^n.s.^
At least one Diagnosis	1298 (88.8)	710 (89.7)	207 (78.9)	527 (83.0)	6.4^n.s.^

* Significant at p ≤ 0.01

^n.s.^Non significant

## Discussion

### Total psychopathology

Since the first psychological autopsy studies by Robins et al. [139] in North America and by Barraclough et al. [7] in Europe, a relatively small number of studies have been carried out. These original studies were descriptive in nature, and only more recently case-control studies have been performed. The data from these studies have consistently suggested a clear relationship between mental disorders and suicide. Here we systematically reviewed these studies and pooled their results whenever possible. Our results show that, on average, 87.3 % of the subjects who committed suicide had a mental disorder. On the other hand, an average of 14.0 % of these subjects was not diagnosed with a psychiatric disorder. A possible explanation is that a diagnosis failed to be detected due to various methodological shortcomings. This possibility is concrete, as psychological autopsy studies rely on informants and/or available medical information to generate diagnostic data. In some cases, the informant has little information on the last weeks or months of life of the subject. Therefore, it is possible that the overall rate of psychopathology observed is still underestimated. This is consistent with findings from recent studies by our group focusing on suicides without an axis I diagnosis [160].

### Specific diagnoses

This review confirms the overall impression from individual studies that affective, substance-related, personality and psychotic disorders account for most of the diagnoses among suicides. The two single most common diagnostic categories among suicide completers were any affective disorders (diagnosed in 43.2 % of suicide cases), and any substance disorders (present in 25.7 % of suicide cases). Recent studies on comorbidity indicate that suicide completers are more likely to have more than one psychiatric diagnosis [142,161]. In a comparison with matched community controls, Foster et al. [142] found a significant increase in suicide risk in the presence of Axis I-Axis II comorbidity (OR = 346.0, p < 0.0001). Our group [161], investigating male completers and controls from the general population, found that suicide cases had an average of 2.36 diagnoses and that comorbidity in completers tended to be of three different patterns, according to mean number of diagnoses (range 1.19 – 4.05) and presence of impulsive-aggressive behaviours. Thus, it would have been interesting to assess overall levels of comorbidity in this review, as well as to investigate what is the amount of overlap between the different diagnoses investigated. However, very little, if any, information about comorbidity was present in the original studies reviewed and this information was impossible to retrieve from the published data.

### Gender differences

The investigation of gender differences in rates of psychopathology associated to suicide should be regarded in light of the methodological limitations of this review, which are primarily related to difficulties in comparing studies carried out using different methodological procedures, diagnostic instruments and criteria, in addition to potential differences in sample characteristics, including age distribution. However, given the important effect that gender seems to have as a suicide risk moderator and the relative lack of appropriate investigation focusing on gender differences in suicide completion, the observed differences in rates of psychopathology in male and female suicides are interesting and should be considered for validation in future studies. Our results indicate that the risk of substance-related disorders, personality disorders and childhood disorders are significantly higher in male suicides, whereas, the risk of affective disorders, specifically, depressive disorders, are greater in female suicides. On average, any substance problems represented 41.8 % (SD 21.1 %) of the male diagnoses and 24.0 % (SD 16.5 %) of the female diagnoses (χ^2 ^7.29 p = 0.007), whereas affective disorders represented 59.4 % (SD 13.9 %) of the female diagnoses and 47.4 % (SD 12.7 %) of the male diagnoses (χ^2 ^2.88 p = 0.089).

Although there has been much discussion on possible factors that could help explain gender differences in suicide rates, most of the studies have primarily focused on psychosocial and demographic risk factors. There is very little data on the possible role of psychiatric and/or behavioural characteristics, which may also mediate gender differences in suicide risk. This study suggests that the underlying psychiatric morbidity may be different in male and female suicide completers. An important question that follows is whether or not the differences found in this study between male and female suicides are the consequence of gender differences in the prevalence of psychiatric disorders in the general population. Although possible, it is unlikely that differences in population rates of psychiatric disorders could explain the different distribution of psychiatric disorders observed in this study, as the gender-specific risks found were not consistently reflecting gender-differences observed in prevalence rates (for instance, schizophrenia and psychotic disorders) and they were not always in the same direction (for instance, personality disorders).

An interesting finding of this study was precisely the absence of gender differences in schizophrenia. This is not necessarily inconsistent with suggestions that most of the suicide cases in schizophrenia are males [162-164], as our findings basically indicate that there are no relative differences between genders in the proportion of suicide cases that are diagnosed with schizophrenia. However, our findings are inconsistent with the common generalization that schizophrenics tend to commit suicide early in the course of the disease because, given gender-differences in the age at onset in schizophrenia [165], with males more likely to have the onset at younger ages, one would expect a considerably higher proportion of schizophrenia among male completers, even if the age distribution in our sample suggests that women in general seemed older than men. In summary, despite the potential methodological limitations discussed above, our results in gender differences in clinical correlates of suicide are interesting and should be further investigated.

### Geographic differences

We also found differences in rates of psychiatric disorders in studies from different geographic origins. This finding may indicate social and cultural factors influencing how one views and interprets suicide and cultural biases towards or against specific diagnoses. Alternatively, as discussed for gender-based comparisons, demographic (age, rural vs. urban samples, socioeconomic and educational level, etc.) differences between the samples could explain some of these results. In view of that, similar limitations, as those for the analysis of gender differences, apply to the analysis of geographical differences in rates of psychopathology associated to suicide (see table 5). American women seem younger than in any other region, Australian women and men appear older than those in the other regions, and no Asian studies provide age means for their sample. In spite of these limitations, our review suggests that, although psychopathology mediates suicide worldwide, there seem to be differences across different parts of the world in the relative proportion of the specific psychiatric disorders found among suicide completers. As mentioned above, these differences may be attributed to variance in psychological autopsy methodologies between countries, or yet, to important differences in the prevalence of psychiatric disorders. Although it is possible that methodological differences between studies play a certain role explaining some of the differences found, it is unlikely that they accounted for all differences found as the studies included in these regional comparisons used similar methods and diagnostic criteria, whereas the differences found were substantial. It is not likely either that diversity between countries in prevalence of psychiatric disorders account for all the observed regional differences, as for some of these disorders, such as schizophrenia, it is thought that there is little variation in prevalence rates between different populations [166]. Thus, the geographical differences observed in the relative proportion of psychiatric disorders among suicide completers is an interesting issue that should be further explored.

Most limitations of this study are common to all quantitative systematic reviews. In particular to this study, one should take into account that the quantitative review was carried out with studies that, although published in a relatively short period of time (from 1986 to 2002), have variation in diagnostic criteria used and have different methodological rigor. Moreover, it is possible that between-study variation in the distribution of a series of demographic variables could have had an impact on our findings. We chose not to control for these methodological differences as given the diverse sources of possible variation, doing so would have considerably limited the number of studies included in the review. Therefore, we opted to be more inclusive and consider the results of this review as preliminary and providing information to be further investigated.

Over the course of this study, a report on another meta-analysis of psychological autopsy studies was published. This study, by Cavanagh et al. [167], reviewed the literature on psychological autopsies and yielded similar overall results. However, there are differences between the study by Cavanagh et al [167] and ours, both in methodology and major aims. While they identified studies through a larger number of library databases, they included only studies up to June 2000. Moreover, they did not investigate risks attributed to specific diagnostic categories, but rather risks attributed to mental health disorders, presence of an affective disorder and comorbidity. They also investigated the role of a few social variables and did not carry out analyses exploring a possible gender and geographic difference in relative rates of psychopathology.

## Conclusions

Our study carried out a systematic review of psychological autopsy studies of suicide and indicates that overall, 87.3% of suicide cases have a history of psychiatric disorders. We also found that male suicides have a different psychiatric profile than female suicide cases and that the relative proportion of psychiatric disorders in suicide completers tends to vary according to geographical region.

## Competing interests

The author(s) declare that they have no competing interests.

## Authors' contributions

GAL carried out the search, extraction of data, analysis and drafted the manuscript. CK helped with the design of the review, and the statistical analysis. GT conceived the study and participated in the design and coordination. All authors read and approved the final manuscript.

## Pre-publication history

The pre-publication history for this paper can be accessed here:



## Supplementary Material

Additional File 1 Table 1 – Mantel-Haenszel Weighed Odds Ratio. This table gives Mantel-Haenszel Weighed Odds Ratio for the 14 case-control studies included in this meta-analysis for the 16 variables of psychiatric disorders. Click here for file
