# Supplementary material for: Psychiatric diagnoses in 3275 suicides: a meta-analysis
Source: BMC Psychiatry. 2004 Nov 4;4:37. doi: 10.1186/1471-244X-4-37 (PMC534107; doi:10.1186/1471-244X-4-37)
Supplement: Additional File 1 — Table 1 – Mantel-Haenszel Weighed Odds Ratio. This table gives Mantel-Haenszel Weighed Odds Ratio for the 14 case-control studies included in this meta-analysis for the 16 variables of psychiatric disorders. [file 1471-244X-4-37-S1.doc]

###### Table 1 – Mantel-Haenszel Weighed Odds Ratio

| **Study (year)** | **Any psychiatric**  **Disorders**  **OR (95% CI)** | **Alcohol problems**  **OR (95% CI)** | **Other substances**  **Problems**  **OR (95% CI)** | **Any substances**  **Problems**  **OR (95% CI)** | **Depressive**  **disorders**  **OR (95% CI)** | **Bipolar disorders**  **OR (95% CI)** | **Any affective disorders**  **OR (95% CI)** |
| --- | --- | --- | --- | --- | --- | --- | --- |
| Appleby et al. (1999)[151] | 26.26 (9.72, 73.73)** | - | - | 10.34 (3.95, 28.19)** | 037 (0.04, 2.43) | - | 3.93 (1.29, 12.80)* |
| Boardman et al. (1999)[152] | 3.11 (2.04, 4.74)** | 1.22 (0.71, 2.08) | 1.11 (0.43, 2.88) | 1.18 (0.75, 1.85) | 2.33 (1.48, 3.70)** | 3.03 (0.28, 76.15) | 2.07 (1.37, 3.13)** |
| Brent et al. (1999)[148] | 14.23 (7.60, 26.86)** | - | - | 12.74 (4.62, 37.95)** | Undefined | - | 8.10 (4.01, 16.62)** |
| Cerel et al. (2000)[155] | 12.16 (2.51, 80.44) | 6.45 (1.89, 21.98)** | 16.08 (3.92, 67.25)** | 8.84 (3.66, 21.32)** | 10.50 (2.76, 39.99)** | 24.88 (2.97, 241.36)** | 11.88 (4.17, 33.65)** |
| Cheng et al. (1995)[16] | 40.98 (9.65, 245.92)** | 2.44 (1.47, 4.04)** | - | 2.44 (1.47, 4.04)** | 5.65 (4.00, 7.99)** | - | 5.65 (4.00, 7.99)** |
| Foster et al. (1999)[142] | 25.38 (11.64, 56.44)** | 5.97 (2.88, 12.56)** | - | 5.59 (3.40, 9.22)** | 10.77 (3.83, 32.69)** | - | 8.80 (3.55, 22.76)** |
| Harwood et al. (2001)[17] | - | - | - | Undefined | 4.01 (1.67, 9.72)** | - | 4.01 (1.67, 9.72)** |
| Hawton et al. (2002)[10] | 123.50 (28.72, 609.04)** | Undefined | Undefined | Undefined | 41.60 (12.55, 147.42)** | Undefined | 55.25 (15.89, 207.64)** |
| Lesage et al. (1994)[150] | 19.30 (6.90, 56.92)** | 3.51 (1.37, 9.38)** | 4.72 (1.62, 14.75)** | 4.02 (1.99, 8.23)** | 10.41 (3.49, 34.87)** | 1.00 (0.21, 4.86) | 5.54 (2.46, 12.95)** |
| Phillips et al. (2002)[9] | 7.98 (5.94, 10.73)** | 1.17 (0.70, 1.97) | - | 1.17 ((0.70, 1.97) | - | - | - |
| Shaffer et al (1996)[18] | 4.84 (2.68, 8.79) | 5.69 ( 1.88, 18.39)** | 15.89 (2.04, 337.33)** | 7.34 (2.81, 20.24)** | 17.77 (6.30, 54.11)** | 0.72 (0.03, 10.25) | 13.05 (5.23, 33.99)** |
| Shaffi et al. (1988)[13] | 18.18 (1.88, 432.46)** | - | - | 4.06 (0.94, 18.51)* | 10.24 (2.06, 56.88)** | - | 10.24 (2.06, 56.88)** |
| Vijayakumar et al. (1999)[159] | 45.05 (18.41, 113.53)** | 5.92 (2.43, 14.91)** | Undefined | 6.47 (2.66, 16.23)** | 15.47 (3.39, 97.88)** | 2.02 (0.14, 57.24) | 11.36 (3.11, 49.10)** |
| Waern et al. (2002)[154] | 122.02 (33.76, 523.33)** | - | - | 56.39 (7.82, 1145.8)** | 23.69 (8.77, 69.35)** | Undefined | 19.23 (8.77, 43.91)** |
| **Total** | **10.50 (9.60, 13.56)**** | **2.53 (2.08, 3.30)**** | **3.72 (2.37, 8.06)**** | **3.51 (3.09, 4.50)**** | **6.23 (5.37, 8.12)**** | **3.03 (1.49, 9.87)**** | **6.00 (5.37, 7.81)**** |

* = p ≤ 0.05; ** = p≤ 0.01

###### Table 1 (continued)

| **Schizophrenia**  **OR (95% CI)** | **Other psychotic**  **disorders or**  **psychoses NOS OR (95% CI)** | **Psychotic disorders and schizophrenia**  **OR (95% CI)** | **Personality**  **Disorders**  **OR (95% CI)** | **Organic**  **Disorders**  **OR (95% CI)** | **Adjustment**  **Disorders**  **OR (95% CI)** | **Anxiety disorders**  **OR (95% CI)** | **Childhood**  **Disorders**  **OR (95% CI)** | **Other disorders OR (95% CI)** |
| --- | --- | --- | --- | --- | --- | --- | --- | --- |
| Undefined | - | Undefined | 6.72 (2.05, 24.34)** | - | 6.63 (1.36, 44.52)** | 0.36 (0.07, 1.70) | - | - |
| 2.38 (0.55, 11.77) | - | 2.38 (0.55, 11.77) | 1.86 (0.94, 3.74) | 0.49 (0.10, 2.25) | - | - | - | - |
| - | - | - | 6.01 (2.66, 13.97)** | - | - | 4.20 (1.43, 13.23)** | - | - |
| - | - | - | 15.31 (1.39, 17.44)** | - | - | 2.66 (0.0, 14.96) | - | - |
| - | - | - | - | - | 0.18 (0.03, 0.80) ** | - | - | - |
| 7.38 (0.89, 11.77) | Undefined | 14.50 (1.93, 301.97)** | 7.53 (3.49, 16.61)** | - | - | 1.56 (0.56, 4.37) | - | - |
| Undefined | - | Undefined | 4.52 (0.82, 32.62) * | 0.21 (0.09, 0.48)** | - | - | - | - |
| Undefined | Undefined | Undefined | 51.08 (6.54, 1082.48)** | - | - | 2.11 (0.41, 9.69)* | - | - |
| Undefined | 2.01 (0.14, 56.69) | 7.19 (0.88, 156.84)* | 2.81 (1.60, 4.96)** | Undefined | 1.71 (0.34, 9.46) | 3.06 (1.03, 9.69)* | 2.06 (1.14, 3.74)** | - |
| 3.56 (1.72, 7.51)** | - | 3.56 (1.72, 7.51)** | - | - | - | - | - | - |
| Undefined | - | Undefined | - | - | - | 2.26 (1.06, 4.83)* | 2.96 (1.14, 3.74)* | Undefined |
| - | - | - | 3.80 (0.56, 32.20) | - | - | - | - | - |
| Undefined | Undefined | Undefined | 8.08 (2.17, 35.54)** | Undefined | 6.68 (1.36, 44.52)** | Undefined | - | 4.13 (0.42, 98.69) |
| - | 13.64 (1.64, 300.26)** | 13.64 (1.64, 300.26)** | - | 0.36 (0.08, 1.40) | - | 4.42 (1.49, 13.69)** | - | 4.72 (0.79, 35.99)** |
| **5.56 (3.12, 10.24)**** | **15.38 (3.53, 97.82)**** | **6.62 (3.94, 11.87)**** | **4.47 (3.53, 6.38)**** | **0.32 (0.17, 0.57)**** | **1.33 (0.72, 2.38)** | **2.43 (1.71, 3.58)**** | **2.37 (1.51, 3.78)**** | **4.98 (1.23, 23.50)**** |

* = p ≤ 0.05; ** = p≤ 0.01
